# Temporal variation in environmental radioactivity and radiation exposure doses in the restricted areas around the Fukushima Daiichi Nuclear Power Plant

**DOI:** 10.1038/s41598-023-49821-8

**Published:** 2023-12-18

**Authors:** Mengjie Liu, Yasuyuki Taira, Masahiko Matsuo, Makiko Orita, Hitomi Matsunaga, Yuya Kashiwazaki, Xu Xiao, Noboru Takamura

**Affiliations:** 1 Department of Global Health, Medicine and Welfare, Atomic Bomb Disease Institute, Nagasaki University Graduate School of Biomedical Sciences, 1-12-4 Sakamoto, Nagasaki City, Nagasaki Prefecture 852-8523 Japan; 2 Fukushima Global Medical Science Center and Radiation Medical Science Center for the Fukushima Health Management Survey Fukushima Medical University, Fukushima City, Fukushima Prefecture Japan

**Keywords:** Environmental sciences, Environmental social sciences, Risk factors

## Abstract

Temporal variation and fluctuation in environmental contamination in Futaba town and Okuma town, the location of the Fukushima Daiichi Nuclear Power Plant (FDNPP), were evaluated based on a car-borne survey conducted from October 2021 to November 2022. Although the environmental radioactivity in the interim storage facility area (ISF) was higher than that in open areas (i.e., the evacuation order lifted areas in Futaba town and the Specific Reconstruction and Regeneration Base area [SRRB] in Okuma town), only minor temporal changes were seen in the ambient dose and detection rate of radiocesium (the proportion of radiocesium detected points per all measuring points) in those areas, respectively. These findings suggest that the observed variations may result from physical decay and environmental remediation. Resuspension caused by human activities and weather could also affect the detection rate of radiocesium. The annual external effective doses in Futaba town and Okuma town were estimated to be at a limited level (< 1 mSv/year). Nevertheless, to help ensure the safety and future prosperity of residents and communities in the affected areas around the FDNPP, long-term follow-up monitoring of temporal exposure dose levels during the recovery and reconstruction phases is extremely important.

## Introduction

On March 11, 2011, the Great East Japan Earthquake (magnitude 9.0) occurred off the east coast of Honshu Island and triggered a massive tsunami that severely affected Iwate, Miyagi, and Fukushima Prefectures, causing a nuclear accident at the Fukushima Daiichi Nuclear Power Plant (FDNPP), located approximately 200 km northeast of Tokyo^1–4^. Immediately after the FDNPP accident, the Japanese government implemented emergency protective measures for the public, such as planning for evacuation, sheltering, and relocation, distributing stable iodine to help prevent further radioactive iodine uptake by the thyroid gland, and restricting food and water consumption. Evacuation of the 20-km area around the FDNPP began immediately on March 11, 2011, and was completed 1 day later. In areas within a 20–30 km radius, residents were ordered to remain indoors and then advised to evacuate voluntarily^4^. Subsequently, NRA, TEPCO and other institutes have continuously conducted a comprehensive monitoring program using airborne survey, car-borne survey, aerial-vehicle survey and radionuclide analysis, including measurements of environmental dose rates and radionuclide activity concentrations in soil, crops, food, and animal feed^2,3,5–7^. Therefore, such survey and monitoring programs are extremely important for the precise evaluation of environmental remediation and the revitalization of Fukushima Prefecture.

By March 19, 2018, the residential areas that had been under evacuation orders, including surrounding roads, residential areas, farmland, and forests, but excluding the difficult to return zones (DRZs), were nearly completely decontaminated^5^. At the same time, an Interim Storage Facility (ISF) was built to store and manage the contaminated soil and waste removed during off-site decontamination work, as well as specified waste (radioactive waste exceeding 8000 Bq/kg) in Fukushima Prefecture, safely until final disposal. Construction of the ISF began in November 2016, and storage of the removed soil and waste began in Okuma town in October 2017 and Futaba town in December 2017^8–11^. Moreover, under the provisions of the Act on Special Measures for the Reconstruction and Revitalization of Fukushima, which was revised in May 2017, six municipalities in restricted areas, including the DRZs in Futaba town and Okuma town, developed revitalization plans^8,10,11^. Under these plans, the Ministry of the Environment conducts decontamination and demolition work in these areas. Due to the efficiency of the decontamination work, on March 4, 2020, the evacuation order was lifted in part of Futaba (evacuation order lifted area)^8^. Subsequently, the evacuation orders for the Specific Reconstruction and Regeneration Base area (SRRB) in Okuma and Futaba were lifted on June 30 and August 30, 2022, respectively^12^.

**Figure 1:**
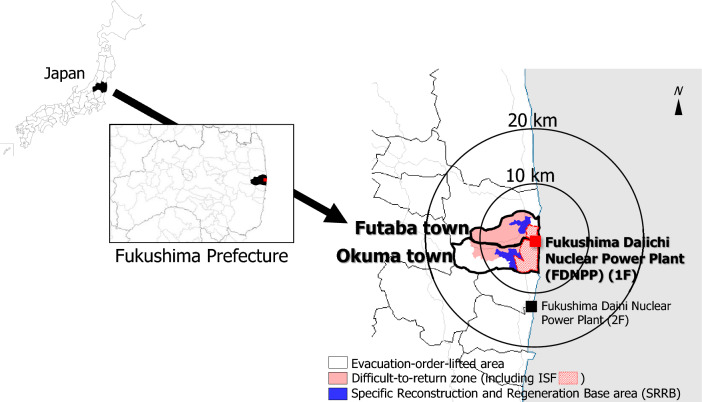
Location of Futaba town and Okuma town, Fukushima Prefecture, Japan. The second author (Y.T.) created the map using GIS software (Green Map III; Tokyo Shoseki, Tokyo, Japan; https://shop.tokyo-shoseki.co.jp/map). Reprinted from Green Map III under a CC BY license, with permission from Tokyo Shoseki; original copyright 2003.

The nuclear accident resulted in the release of various artificial radionuclides, including cesium-134 (^134^Cs), cesium-137 (^137^Cs), and iodine-131 (^131^I) into the atmosphere and eventual deposition on land and sea in the areas around the FDNPP^1–3^. Radionuclides with long half-lives such as ^134^Cs (half-life: 2.1 years) and ^137^Cs (half-life: 30 years) remain in the environment, although more than 12 years have passed in March 2023 since the FDNPP accident, which was one source of the ambient dose rate^1,4,6^. Although environmental remediation in the SRRB might allow new residential areas to be constructed in Futaba town and Okuma town in the future, residents in Fukushima still have concerns about radiation which affects their intention to return^13,14^. In addition, environmental radioactivity around the restricted areas, including the ISF, should be monitored and controlled because these areas are next to the SRRB. Therefore, in the present study, a car-borne survey was conducted to evaluate temporal variation in ambient dose and detection rate of radiocesium in Futaba town and Okuma town (Fig. 1).

## Results

**Figure 2:**
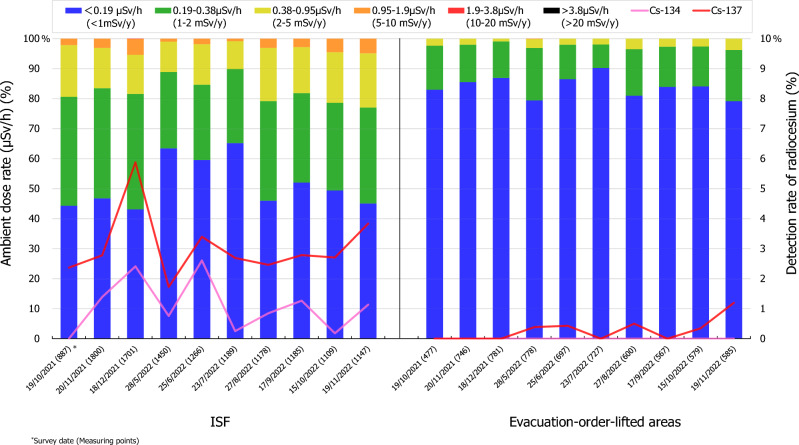
Temporal variation in the ambient dose rate distribution and detection rate of radiocesium in Futaba town.

**Figure 3:**
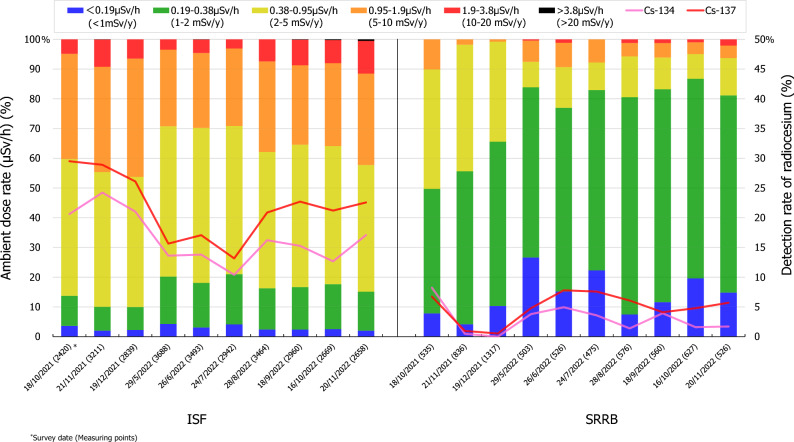
Temporal variation in the ambient dose rate distribution and detection rate of radiocesium in Okuma town.

In the present study, 10 surveys were conducted in Futaba town and Okuma town from October 2021 to November 2022. The frequency distributions of the ambient dose rate and detection rate of radiocesium (^134^Cs and ^137^Cs) within the ISF, SRRB, and evacuation order lifted areas of Futaba town and Okuma town are shown in Tables 1 and 2 and Figs. 2 and 3. The median ambient dose rate in the ISF and evacuation order lifted areas in Futaba town ranged from 0.15 to 0.21 μSv/h (measurement points are 887–1800) and from 0.053 to 0.086 μSv/h (477–781), respectively, and those in the ISF and SRRB in Okuma town ranged from 0.68 to 0.90 μSv/h (2420–3688) and from 0.23 to 0.38 μSv/h (503–1317), respectively (Tables 1 and 2). Moreover, the ambient dose rate in the ISF areas (> 0.95 μSv/h) were significantly higher than those in the evacuation order lifted areas in Futaba town and the SRRB in Okuma town (*p* < 0.01). The proportions of the ISF area exceeding this value (> 0.95 μSv/h) were approximately 1–5% in Futaba town and 29–46% in Okuma town, and approximately 0% and 1–10% in the evacuation order lifted areas and SRRB, respectively (Figs. 2 and 3). The ambient dose rate in the ISF area clustered < 0.38 μSv/h in Futaba town (79–89%) and 0.38–1.9 μSv/h in Okuma town (73–83%). By contrast, the ambient dose rate clustered < 0.19 μSv/h (79–90%) in the evacuation order lifted areas in Futaba town and 0.19–0.95 μSv/h (66–94%) in the SRRB in Okuma town.

**Table 1:** Ambient dose rate in the interim storage facility (ISF) area and evacuation order lifted area in Futaba town from October 2021 to November 2022.

Survey date	ISF		Evacuation order lifted area	
	Measurement points	Median (min–max) (μSv/h)	Measuring points	Median (min–max) (μSv/h)
19/10/2021	887	0.21 (0.050–1.8)	477	0.059 (0.029–0.63)
20/11/2021	1800	0.20 (0.048–1.8)	746	0.062 (0.029–0.60)
18/12/2021	1701	0.21 (0.039–2.0)	781	0.064 (0.029–0.51)
28/5/2022	1450	0.16 (0.042–1.3)	778	0.086 (0.025–1.0)
25/6/2022	1266	0.16 (0.032–1.3)	697	0.060 (0.025–0.71)
23/7/2022	1189	0.15 (0.040–1.3)	727	0.053 (0.020–0.49)
27/8/2022	1178	0.21 (0.034–1.6)	600	0.070 (0.030–0.68)
17/9/2022	1185	0.19 (0.035–1.5)	567	0.067 (0.027–0.82)
15/10/2022	1109	0.19 (0.040–1.5)	579	0.083 (0.024–0.72)
19/11/2022	1147	0.21 (0.049–1.9)	585	0.083 (0.030–0.85)
Annual estimated effective doses of *workers* in mSv/year (average)		0.17	Annual estimated effective doses of *residents* in mSv/year (average)	0.091

**Table 2:** Ambient dose rate in the interim storage facility (ISF) area and Specific Reconstruction and Regeneration Base (SRRB) area in Okuma town from October 2021 to November 2022.

Survey date	ISF		SRRB	
	Measurement points	Median (min–max) (μSv/h)	Measuring points	Median (min–max) (μSv/h)
18/10/2021	2420	0.83 (0.11–3.4)	535	0.38 (0.13–1.3)
21/11/2021	3211	0.86 (0.11–4.0)	836	0.35 (0.15–1.3)
19/12/2021	2839	0.90 (0.10–3.4)	1317	0.32 (0.081–1.3)
29/5/2022	3688	0.69 (0.10–3.3)	503	0.23 (0.12–2.0)
26/6/2022	3493	0.68 (0.10–4.1)	526	0.25 (0.12–2.1)
24/7/2022	2484	0.69 (0.10–2.9)	942	0.24 (0.11–1.9)
28/8/2022	3464	0.77 (0.11–3.7)	576	0.27 (0.15–2.2)
18/9/2022	2960	0.75 (0.10–4.2)	560	0.26 (0.11–2.2)
16/10/2022	2669	0.73 (0.094–5.1)	627	0.24 (0.12–2.0)
20/11/2022	2658	0.82 (0.094–4.9)	526	0.25 (0.11–2.4)
Annual estimated effective doses of *workers* in mSv/year (average)		0.84	Annual estimated effective doses of *residents* in mSv/year (average)	0.76

In other words, the ambient dose rate (> 0.38 μSv/h) in the ISF areas showed a significantly higher distribution (*p* < 0.01) in Okuma town (80–90%) than in Futaba town (10–23%). On the other hand, the ambient dose rate in the SRRB in Okuma town (0.38 μSv/h; 59–87%) was higher than that in the evacuation order lifted areas in Futaba town (< 0.19 μSv/h; 79–90%) (Figs. 2 and 3). The ambient dose rate has stabilized at the relatively low level in Futaba town and Okuma town, although there were some fluctuations during the survey period (Figs. 2 and 3). For example, the median ambient dose rate in the SRRB in Okuma town seems decreased by 34%, from 0.38 μSv/h in October 2021 to 0.25 μSv/h in November 2022, in Futaba town seems increased from 0.059 μSv/h in October 2021 to 0.083 μSv/h in November 2022, and the ISF areas showed relatively lower values in May, June, and July 2022 in both towns (Tables 1 and 2).

Furthermore, the detection rate of radiocesium (^134^Cs and ^137^Cs) indicated the proportion of measurement points where ^134^Cs and ^137^Cs could be detected compared to all measurement points in Futaba town and Okuma town as shown in Figs. 2 and 3. The detection rate of ^134^Cs and ^137^Cs ranged from 0 to 2.6% and from 1.7 to 5.9% in Futaba town, respectively, and from 10.4 to 24.2% and from 13.2 to 29.5% in Okuma town, respectively. The detection rate of radiocesium in Okuma town were significantly higher than those in Futaba town (p < 0.01). Additionally, the value in the evacuation order lifted area and SRRB were lower compared to those for the ISF area, with the detection rate of ^137^Cs ranging from 0 to 1.2% in Futaba town (^134^Cs no detected points), and the detection rate of ^134^Cs ranging from 0 to 8.2% and the detection rate of ^137^Cs ranging from 0.5 to 7.8% in Okuma town **(**Figs. 2 and 3 and Supplementary Figs. S1, S2 and S3). The detection rate of ^134^Cs were lower than those of ^137^Cs because 12 years had passed since the FDNPP accident (approximately six times the physical half-life of ^134^Cs). The physical decay of radiocesium was estimated to be 4.9% from October 2021 to November 2022, which contributed to the decrease of the detection rate of radiocesium and ambient dose rate in Futaba town and Okuma town, respectively (Supplementary Table S1).

The annual external effective doses for decontamination workers in the ISF were estimated at 0.17 mSv/year in Futaba town and 0.84 mSv/year in Okuma town. By contrast, the annual effective doses (corresponding to indoor and outdoor activities of daily living) for residents who might return to the evacuation order lifted areas and SRRB were estimated at 0.091 mSv/year in Futaba town and 0.76 mSv/year in Okuma town (Supplementary Tables S2 and S3, Figs. S2 and S3).

## Discussion

Construction of the ISF in Futaba town and Okuma town began in 2017, with storage facility for removed soil set up in three areas in Futaba town and five areas in Okuma town^9–11,15^. The total capacity for contaminated soil was 3.1 million m^3^ in Futaba town and 10 million m^3^ in Okuma town. As of March 2023, two storage facilities in Futaba town and one in Okuma town have been completed^15^. During the construction of ISF, radioactive waste is covered with soil or concrete as a protective layer to prevent the spread of radioactive materials, while to prevent the dispersion of radioactive materials when bringing them into ISF, the radioactive waste is carried in flexible containers during transportation^9,15,16^. In the present study, the stable ambient dose in ISF suggested that there was no obvious leakage or scattering of radioactive material in the ISF area during the construction process in our survey period. From October 2021 to November 2022, the ambient dose rate and detection rate of radiocesium in Okuma town were significantly higher in Futaba town (*p* < 0.001). Immediately after the FDNPP accident, various radionuclides were released from around the FDNPP into the atmosphere, eventually being deposited on land and at sea in the surrounding areas^5^. Due to the influence of terrain and weather, the amounts of artificial radionuclides released from nuclear reactors and diffusion scales absolutely differed between Futaba town and Okuma town^4,8,17,18^. The databases of NRA's car-borne survey showed that the ambient dose rate in Okuma town was significantly higher than that in Futaba town after the accident^18^. Therefore, the difference in the initial contamination in Futaba town and Okuma town are considered to provide a direct reflection of the ambient dose rate and detection rate of radiocesium, even after the decontamination process, because whole contamination materials such as deposits, soil, and trees were not completely removed in the forest near the town^8,10,11^. Moreover, the ISF remains a restricted area and decontamination work has not yet been carried out, which contributed to the higher environmental radioactivity value in Okuma town. Our results show that the average ambient dose in Okuma town and Futaba town during the survey period were 0.37 μSv/h and 0.11 μSv/h in the SRRB and evacuation order lifting areas. In the ISF the value was 0.91 μSv/h and 0.27 μSv/h in Okuma town and Futaba town, respectively. The ambient dose rate and detection rate of radiocesium were significantly higher in the ISF than in the evacuation order lifted area and SRRB in Futaba town and Okuma town (p < 0.001). In general, the efficient and thorough decontamination processes can reduce ambient dose by removing radioactive materials from affected areas (environmental remediation)^19–24^. In our previous study in Tomioka town, Fukushima Prefecture, a significant difference in ambient dose rate since 2017 was observed between the decontaminated and non-decontaminated areas^19–21^. The ambient dose rate in the decontaminated area decreased by 71.9% (from 1.0 to 0.32 μSv/h during 2018–2019)^19^. According to another report, the average ambient dose rate in decontaminated locations was about 20% lower than that in non-decontaminated areas^22^. Reports from the Ministry of the Environment showed decontamination reducing the ambient dose rate by 60% at 1 m above ground level in residential areas and by 42% on roads^8,23^. In Futaba town and Okuma town, the decontamination processes at the SRRB started on September 12, 2017 and March 9, 2018, respectively, mainly targeting public facilities such as station square, nurseries, and gymnasium^8,10–12^. The decontamination process in the evacuation order lifted area in Futaba town started much earlier than that at the SRRB and was completed in March 2016, well before the evacuation order was lifted on March 4, 2020^8^. Additionally, towards the goal of lifting evacuation order at entire SRRB in Spring of 2022–2023, the Ministry of the Environment is conducting demolition and decontamination cooperating with Futaba town and Okuma town during our survey periods^8^. Therefore, the ambient dose rates in SRRB decreased from 0.38 μSv/h in October 2021 to 0.23 μSv/h in May 2022 in Okuma Town, which has been maintained at a relatively low level since then can be considered the benefit of decontamination. Moreover, the environmental radioactivity in the evacuation order lifted areas in Futaba town has been continuously stabilized at extremely low levels as a result of decontamination as well, despite some fluctuations being noticed. Although we noticed a smaller ambient dose rate in May, June, and July 2022 in the ISF areas, it is difficult to clarify the relationship between season and ambient dose rate. We believe that the smaller values observed during this period may be related to the precipitation brought by the seasonal rainfall in this area from May to July^25–27^. Precipitation increases soil moisture, and higher water content in the soil limits gamma-ray emission^26^. The wash-out caused by precipitation can also cause surface radioactive materials to migrate to deeper layers, thereby reducing observed ambient dose rates^27^.

On the other hand, although the detection rate of radiocesium (mainly ^137^Cs) fluctuated in a small range throughout the survey period, it always remained at a relatively low level. In our previous study, accident‐derived ^137^Cs levels in the SRRB in Tomioka were observed in airborne dust samples, which suggested that the ^137^Cs radioactivity in the airborne dust was primarily associated with particles that were resuspended by localized winds and the transfer of construction vehicles as opposed to the decontamination and demolition operations (Supplementary Table S4)^28^. Furthermore, human activities such as the transportation of contaminants (removal of soil and radioactive waste) and land restoration might have caused some fluctuations in the ambient dose rate^19,28^. In the present study, our result also suggested that the fluctuations in ambient dose rate caused by human activities are accompanied by fluctuations in the detection rate of radiocesium. According to another report, weekly changes in vehicular traffic tends to affect the accumulation of airborne dust particles and radioactive materials resuspended in the air, thereby contributing to temporary variation in the concentration of radiocesium^29^. In addition, wind direction, wind speed, and other meteorological factors can also cause changes in radiocesium concentrations^27,30–32^. According to previous studies in Fukushima, the wind can affect the deposition level of ^137^Cs, with high concentrations in the air associated with areas of high ^137^Cs deposition^30^. Radiocesium resuspension and deposition can also be influenced by meteorological events such as rain out (washout), which can transfer radiocesium in the surface layer to the lower layer^27^. According to reports of the Chernobyl accident, a positive relationship was found between airborne radiocesium concentrations and wind speed^31,32^. In the present study, the results indicated that the detected radiocesium came not only from materials release from FDNPP accident but also from subsequent airborne radiocesium resuspended at certain locations during the study period.

The estimated annual effective doses for decontamination workers and residents of the decontamination area were lower than the recommended limit set by the Japanese government based on the recommendation of the International Commission on Radiological Protection^33–35^ (Supplementary Tables S2 and S3, Figs. S2 and S3). Nevertheless, to control artificial radioactivity, avoid unnecessary radiation exposure, and alleviate radiation anxiety due to the FDNPP accident in these areas, environmental radioactivity monitoring and special education, including radiation safety for workers who engage in decontamination work and residents who will return to the SRRB, are necessary.

In the present study, changes in the ambient dose rate by season and weather were difficult to identify through a horizontal comparison based on car-borne surveys. Moreover, the detection rate of radiocesium in this study mainly comes from radiocesium deposited in the surface soils or asphalt of roads, trees, and plants, which is obviously lower than that of soil samples. However, the main artificial radionuclides derived from the FDNPP accident, such as ^137^Cs, could be analyzed to sufficiently precise levels using high-purity germanium detectors. Therefore, the combination of radionuclide analysis of environmental samples such as soil and extensive monitoring via a car-borne survey could accurately evaluate the decontamination effects and external and internal exposure levels. These findings suggest that long-term follow-up monitoring is extremely important for the reconstruction of affected areas, including the SRRB, around the FDNPP.

## Materials and methods

### Survey location

The FDNPP (37°25′ N, 141°02′ E) is located on the boundary between Futaba town (37°25′ N, 141°02′ E) and Okuma town (37°25′ N, 141°02′ E) in Fukushima Prefecture on the east coast of Honshu Island, Japan. Both towns include DRZ and SRRB areas (Fig. 1)^4^. Further decontamination efforts have been continuing in the SRRB of Futaba town and Okuma town.

### Survey of ambient dose rate and radiocesium detection

The ISF within the DRZ and SRRB areas (including the evacuation order lifted area) of Futaba town and Okuma town was surveyed using the Radi-probe car-borne survey system (Chiyoda Technology Corp., Tokyo, Japan) connected to a handheld radiation detector (HDS-101GN; Mirion Technologies, Inc., Japan) from October 2021 to November 2022 (10 times in the ISF, SRRB, and evacuation-over-lifted area of Futaba town and Okuma town)^36,37^. Radi-Probe is a data acquisition system for a car-borne survey. The system consists of a handheld radiation detector, a GPS receiver, a micro-camera, and a laptop personal computer (PC) that controls all devices^36^. The entire system is installed on the sedan car, with the handheld radiation detector was set on the front passenger seat about 1 m above the ground. The dose rate and the gamma-ray energy spectra obtained by the handheld radiation detector, tagged with the GPS position are continuously stored in the PC, which automatically captures position coordinates and a photo every 5 s in addition to spectral segments every 0.2 seconds^36,37^. The handheld radiation detector is a large thallium-doped cesium iodide scintillator with high sensitivity (typical 1400 cps per µSv/h for the ^137^Cs source), and the measurable dose-rate range is 10 nSv/h–100 μSv/h, the measurable energy range gamma-ray is 30 keV–3 MeV using a multichannel analyzer with 512 channels^37^. The measured spectrum is internally converted to dose rate^36,37^. In other words, we measured gamma-ray directly by the standard method using the scintillator (conversion gamma-ray signal to electrical signals). The Radi-probe system shows the detected energy peaks of radiocesium registered in the detected net count values and their associated confidence (with levels 1–10 used as reference values)^19,38^. In other words, the detected rate of radiocesium was obtained qualitatively from the region of interest of the energy peaks of radiocesium (604 keV for ^134^Cs and 662 keV for ^137^Cs), which can be used to indicate the qualitatively surface deposition concentration of radiocesium, with values 1–10 used as reference confidence interval levels. Software installed on the PC has a graphical interface that can display gamma-ray energy spectra and a map with color-scaled ambient dose equivalent rates. Temporal variation of the ambient dose equivalent rate is also displayed on the graphical interface. Snapshots taken by the micro-camera in the front of the car are also displayed, which confirm the geographic environment and weather^36,37^. The calibration of Radi-probe system is carried out by Chiyoda Technol every 2 years. Additionally, we carried out the easy efficiency calibration by the standard radiation source (Japan Radioisotope Association, Tokyo, Japan) before each survey. Generally, the chassis and walls of a vehicle provide a shielding effect against external radiation; numerous other factors, such as the type of vehicle and the number of occupants, also affect this shielding factor^20^. Therefore, the shielding effect was calculated by measuring the interior and exterior of the vehicle in an open and flat area at a height of 1 m above the ground before each monitoring session. During the survey periods, the sedan car was driven at a constant speed by the same person, the shielding factors ranged from 1.19 to 1.84.

### Effective dose

The effective dose through external exposure is calculated using the following formula^19^:

1 $$E_{i} = \, (D_{out} {-}D_{BG} ) \cdot T \cdot R$$

2 $$E_{w} = \mathop \sum \limits_{i = 1}^{12} E_{i}$$

3 $$E = E_{out} + E_{in}$$

4 $$E_{out/in} = \, \left( {D_{out/in} {-}D_{BG} } \right) \cdot T \cdot F \cdot R$$

5 $$D_{in} = r \cdot D_{out}$$

where *E*_*i*_ is the estimated external effective dose (mSv/month by median), *E*_*w*_ the external effective dose for decontamination workers (mSv/year), *E* the external effective dose for residents who are going to return to the decontaminated area (mSv/year), *E*_*out/in*_ the external effective dose for outdoor and indoor workers, *D*_*out/in*_ the dose rate for a height of 1 m above ground outside and inside the house (μSv/h), *D*_*BG*_ 0.04 μSv/h, which was measured in the area of interest before the accident^39^, *T* the work time (240 days × 8 h; the normal labor standard in Japan), *F* the occupancy factor (with 16 and 8 h [24 h/day] considered to represent the indoor and outdoor activities of daily living, respectively, based on the guideline of the Ministry of the Environment, Japan), *R* the age-dependent dose conversion coefficient for adults (0.6 corresponds to the effective dose for an adult), and *r* the deposited gamma location factor for a wooden house (0.4)^40^.

### Data analysis

All data followed a non-normal distribution. To compare differences among the measurement areas in the same period and the temporal variation within the same areas, the data were analyzed using the Mann–Whitney *U* and Kruskal–Wallis *H* tests.

### Supplementary Information

Supplementary Figure S1.

Supplementary Information 2.

Supplementary Information 3.

## Data Availability

The datasets used and analyzed during the current study are available from the corresponding author upon reasonable request.
