# Supplementary material for: Temporal variation in environmental radioactivity and radiation exposure doses in the restricted areas around the Fukushima Daiichi Nuclear Power Plant
Source: Sci Rep. 2023 Dec 18;13:22459. doi: 10.1038/s41598-023-49821-8 (PMC10725873; doi:10.1038/s41598-023-49821-8)
Supplement: Supplementary file 1 — Supplementary Figure S1. [file 41598_2023_49821_MOESM1_ESM.pdf]

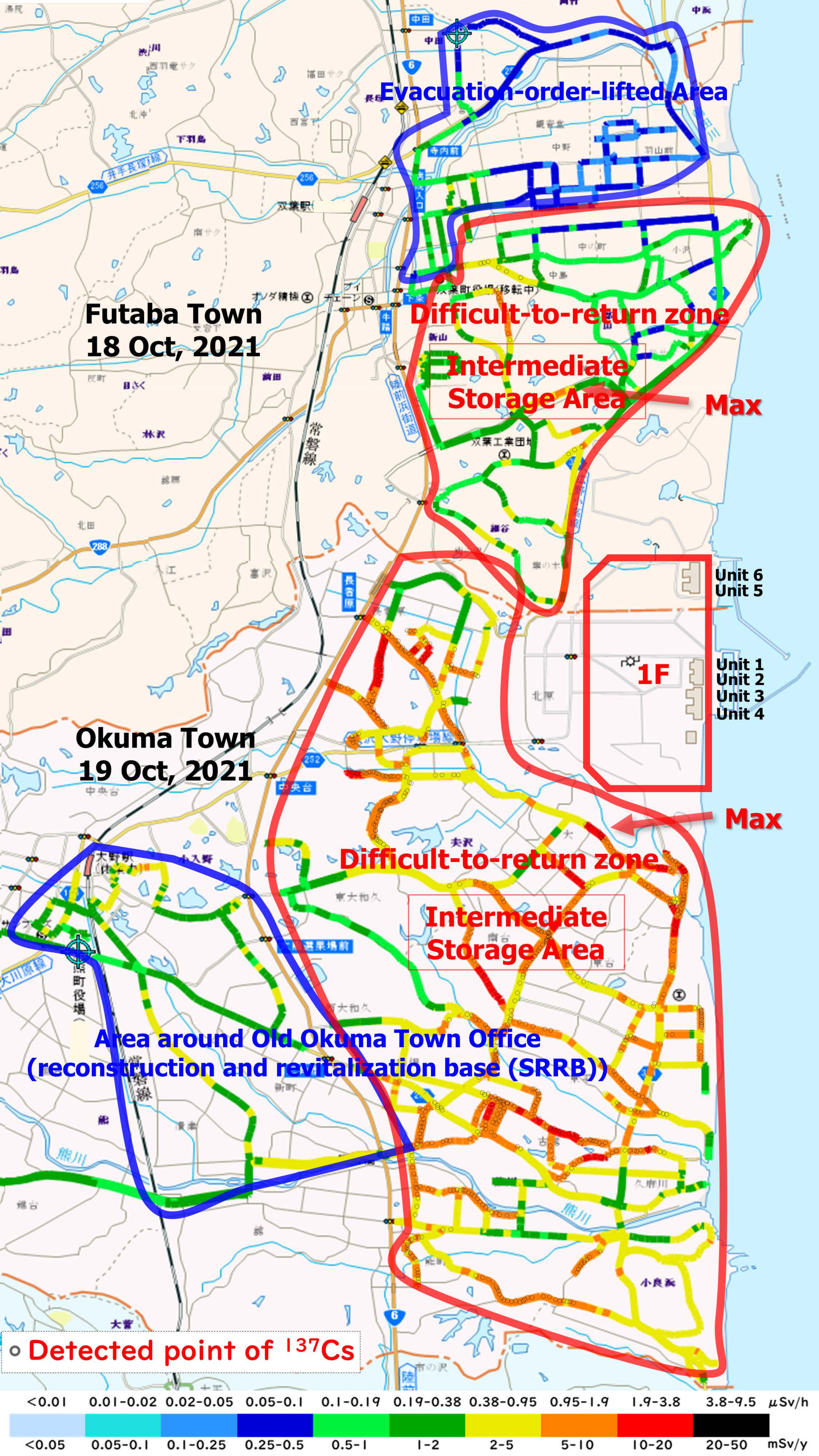

Evacuation-order-lifted Area
Futaba Town
18 Oct, 2021
Difficult-to-return zone
Intermediate Storage Area
Max
Unit 6
Unit 5
Unit 1
Unit 2
Unit 3
Unit 4
1F
Okuma Town
19 Oct, 2021
Max
Difficult-to-return zone
Intermediate Storage Area
Area around Old Okuma Town Office
(reconstruction and revitalization base (SRRB))
Detected point of ¹³⁷Cs
<0.01
0.01-0.02
0.02-0.05
0.05-0.1
0.1-0.19
0.19-0.38
0.38-0.95
0.95-1.9
1.9-3.8
3.8-9.5
μSv/h
<0.05
0.05-0.1
0.1-0.25
0.25-0.5
0.5-1
1-2
2-5
5-10
10-20
20-50
mSv/y

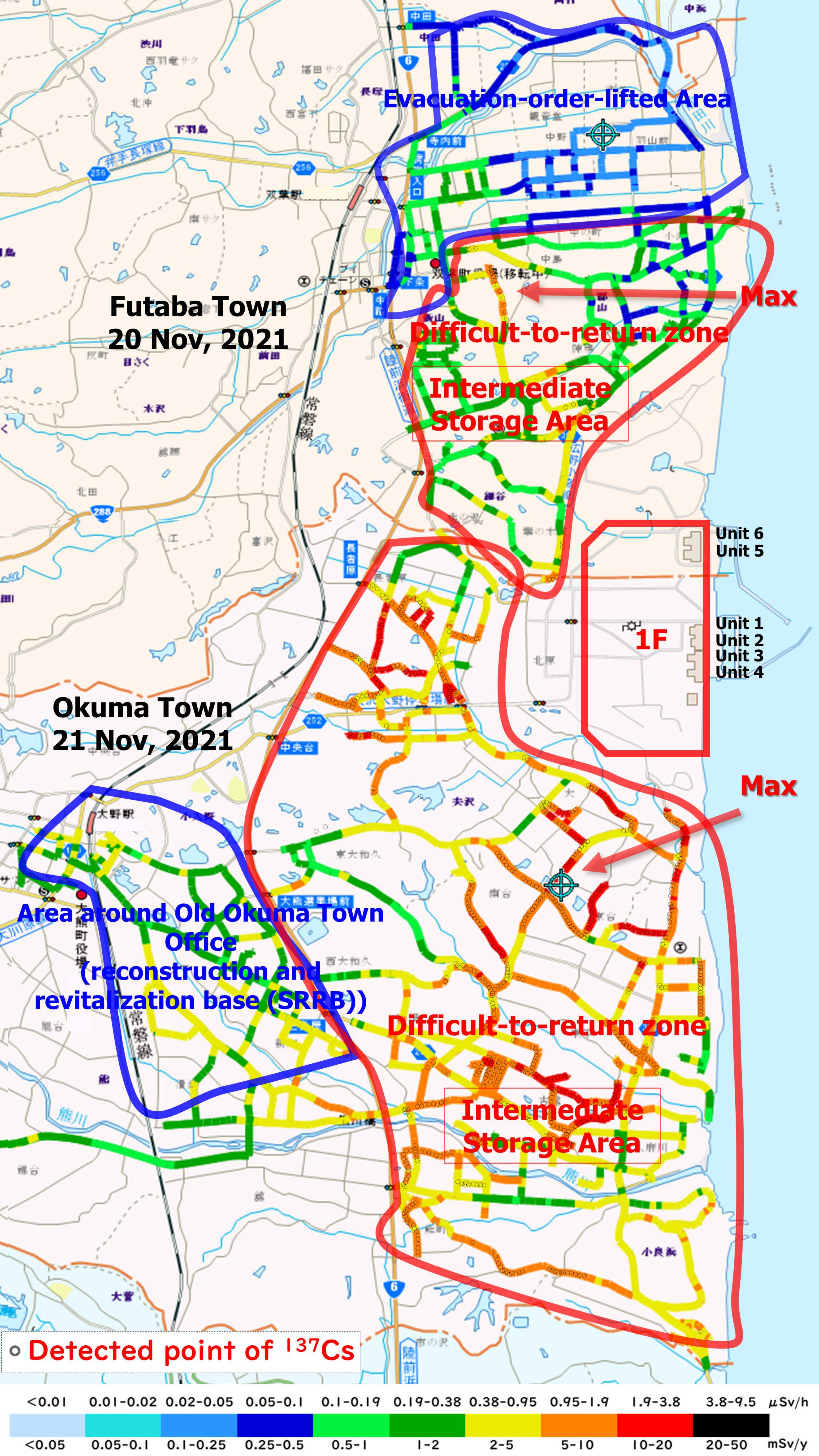
Evacuation-order-lifted Area
Max
Difficult-to-return zone
Intermediate Storage Area
Futaba Town
20 Nov, 2021
Unit 6
Unit 5
Unit 1
Unit 2
Unit 3
Unit 4
1F
Okuma Town
21 Nov, 2021
Max
Area around Old Okuma Town Office (reconstruction and revitalization base (SRRB))
Difficult-to-return zone
Intermediate Storage Area
Detected point of ^{137}Cs
<0.01 0.01-0.02 0.02-0.05 0.05-0.1 0.1-0.19 0.19-0.38 0.38-0.95 0.95-1.9 1.9-3.8 3.8-9.5 μSv/h
<0.05 0.05-0.1 0.1-0.25 0.25-0.5 0.5-1 1-2 2-5 5-10 10-20 20-50 mSv/y

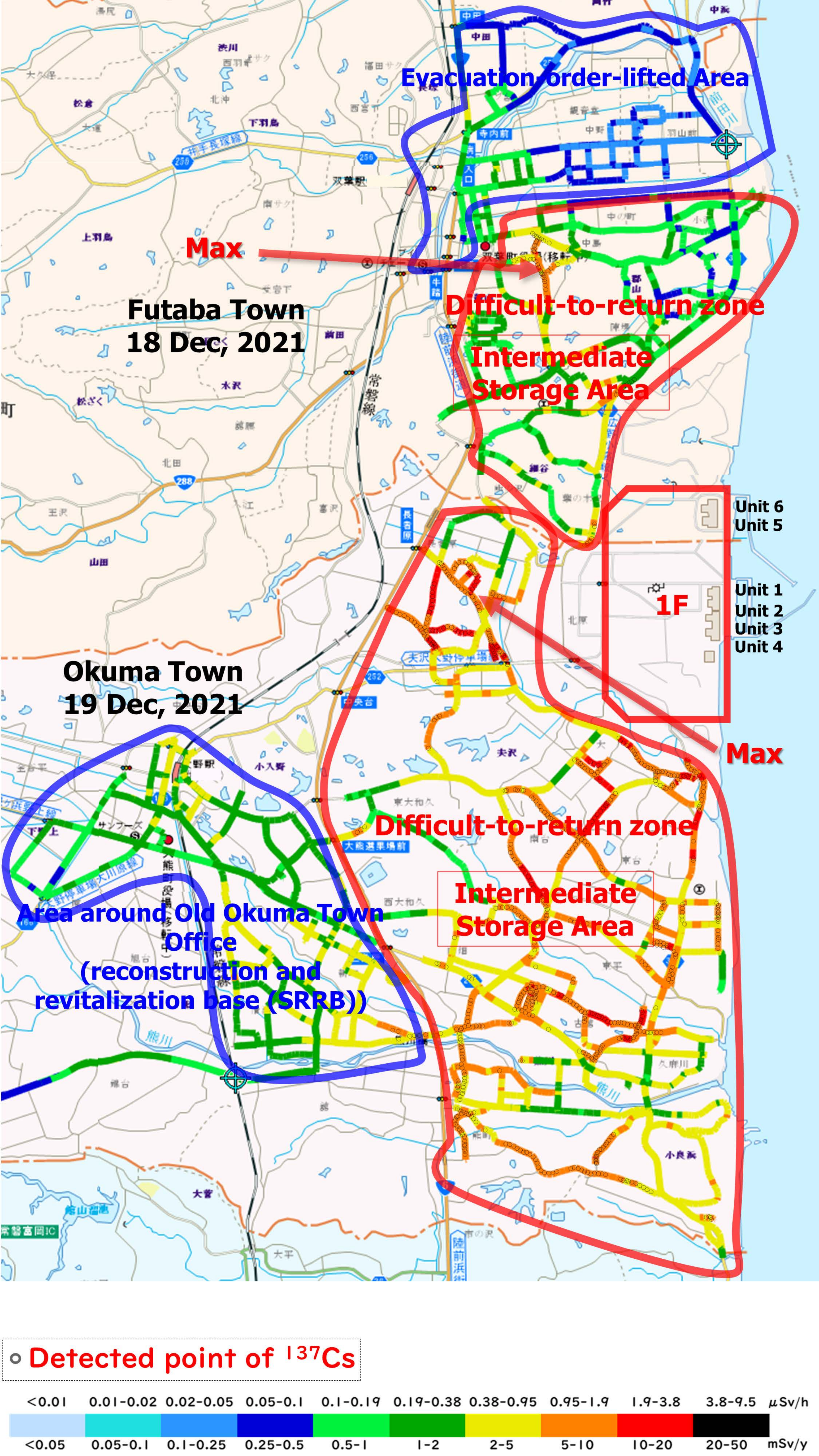
Evacuation order-lifted Area
Max
Difficult-to-return zone
Intermediate Storage Area
Futaba Town
18 Dec, 2021
Unit 6
Unit 5
Unit 1
Unit 2
Unit 3
Unit 4
1F
Okuma Town
19 Dec, 2021
Max
Difficult-to-return zone
Intermediate Storage Area
Area around Old Okuma Town Office (reconstruction and revitalization base (SRRB))
Detected point of 137Cs
<0.01 0.01-0.02 0.02-0.05 0.05-0.1 0.1-0.19 0.19-0.38 0.38-0.95 0.95-1.9 1.9-3.8 3.8-9.5 μSv/h
<0.05 0.05-0.1 0.1-0.25 0.25-0.5 0.5-1 1-2 2-5 5-10 10-20 20-50 mSv/y

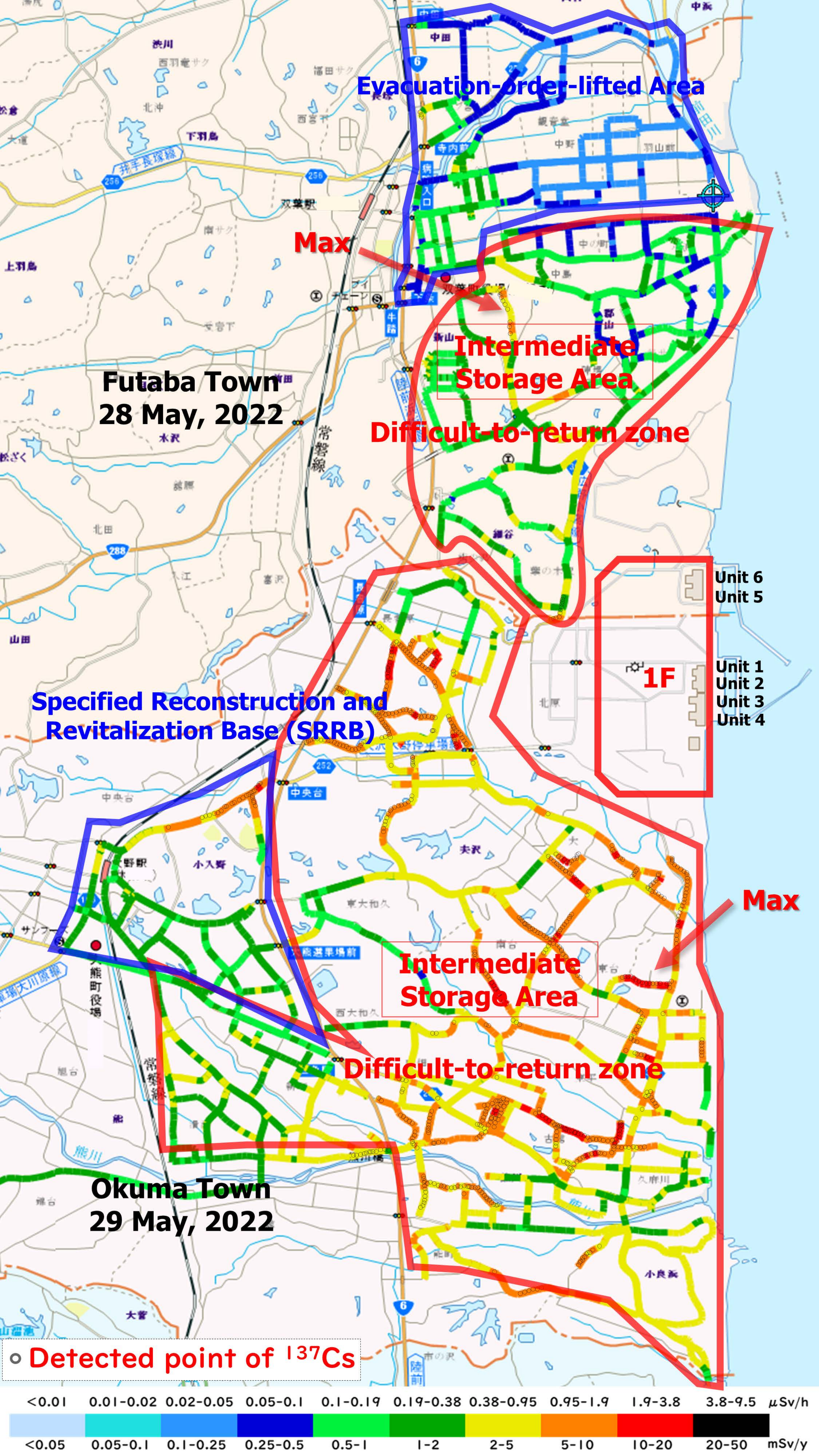
Evacuation-order-lifted Area
Max
Intermediate Storage Area
Difficult-to-return zone
Futaba Town
28 May, 2022
Unit 6
Unit 5
1F
Unit 1
Unit 2
Unit 3
Unit 4
Specified Reconstruction and Revitalization Base (SRRB)
Max
Intermediate Storage Area
Difficult-to-return zone
Okuma Town
29 May, 2022
Detected point of 137Cs
<0.01 0.01-0.02 0.02-0.05 0.05-0.1 0.1-0.19 0.19-0.38 0.38-0.95 0.95-1.9 1.9-3.8 3.8-9.5 μSv/h
<0.05 0.05-0.1 0.1-0.25 0.25-0.5 0.5-1 1-2 2-5 5-10 10-20 20-50 mSv/y

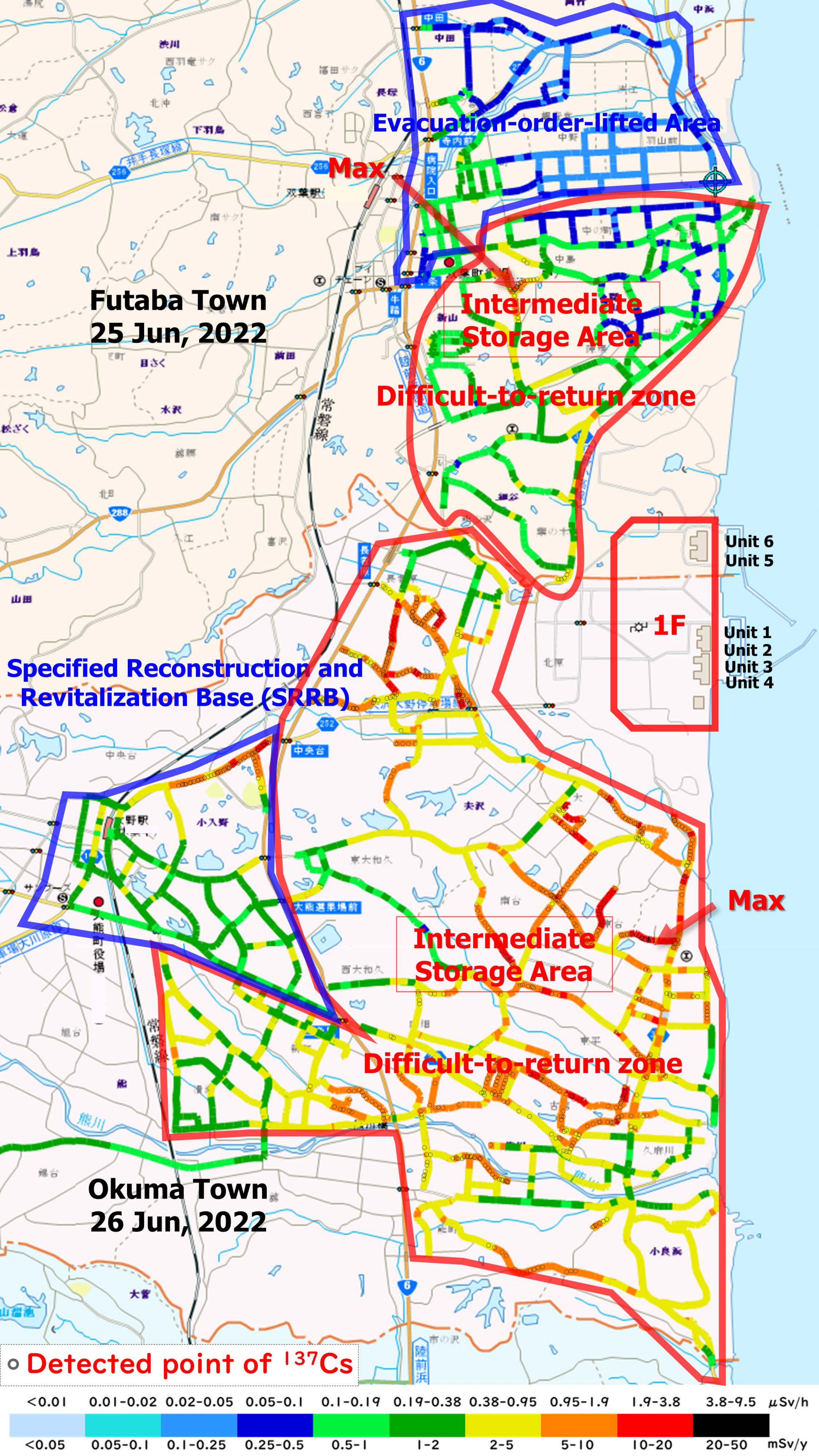

Evacuation-order-lifted Area
Max
Futaba Town
25 Jun, 2022
Intermediate Storage Area
Difficult-to-return zone
Unit 6
Unit 5
1F
Unit 1
Unit 2
Unit 3
Unit 4
Specified Reconstruction and Revitalization Base (SRRB)
Max
Intermediate Storage Area
Difficult-to-return zone
Okuma Town
26 Jun, 2022
Detected point of 137Cs
<0.01 0.01-0.02 0.02-0.05 0.05-0.1 0.1-0.19 0.19-0.38 0.38-0.95 0.95-1.9 1.9-3.8 3.8-9.5 μSv/h
<0.05 0.05-0.1 0.1-0.25 0.25-0.5 0.5-1 1-2 2-5 5-10 10-20 20-50 mSv/y

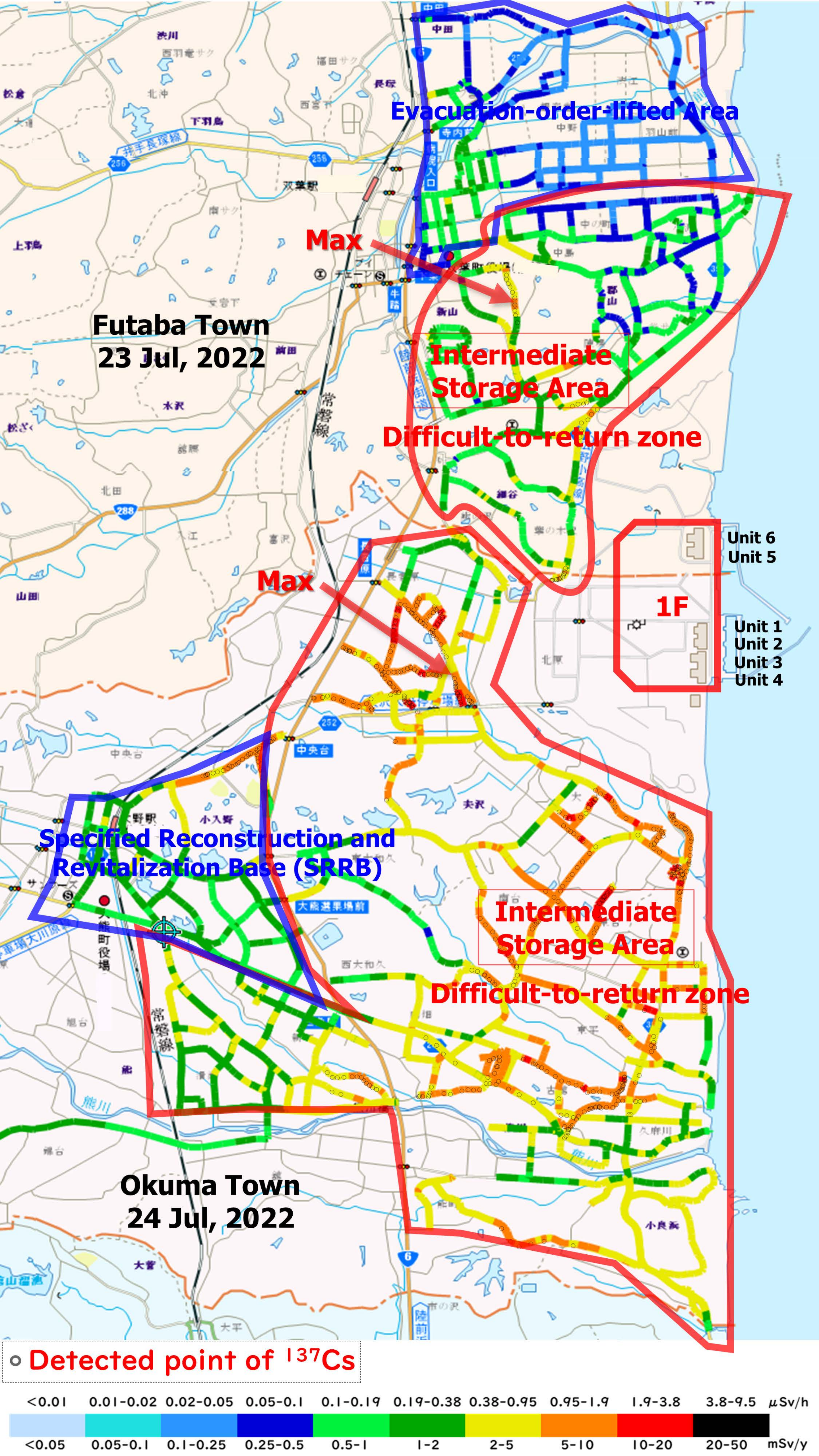
Evacuation-order-lifted Area
Max
Futaba Town
23 Jul, 2022
Intermediate Storage Area
Difficult-to-return zone
Max
Unit 6
Unit 5
1F
Unit 1
Unit 2
Unit 3
Unit 4
Specified Reconstruction and Revitalization Base (SRRB)
Intermediate Storage Area
Difficult-to-return zone
Okuma Town
24 Jul, 2022
Detected point of ^{137}Cs
<0.01 0.01-0.02 0.02-0.05 0.05-0.1 0.1-0.19 0.19-0.38 0.38-0.95 0.95-1.9 1.9-3.8 3.8-9.5 μSv/h
<0.05 0.05-0.1 0.1-0.25 0.25-0.5 0.5-1 1-2 2-5 5-10 10-20 20-50 mSv/y

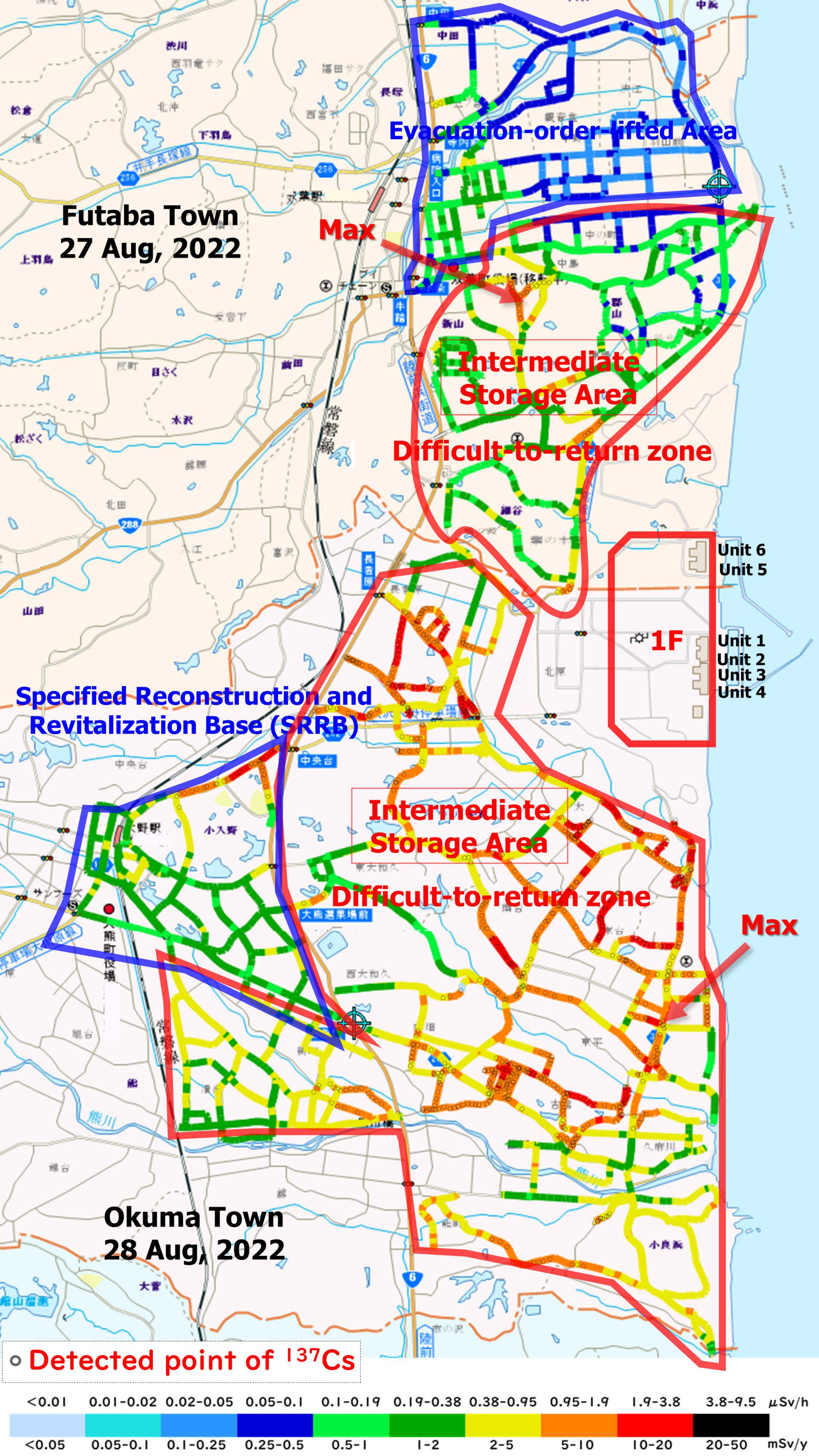
Evacuation-order-lifted Area
Futaba Town
27 Aug, 2022
Max
Intermediate Storage Area
Difficult-to-return zone
Unit 6
Unit 5
1F
Unit 1
Unit 2
Unit 3
Unit 4
Specified Reconstruction and Revitalization Base (SRRB)
Intermediate Storage Area
Difficult-to-return zone
Max
Okuma Town
28 Aug, 2022
Detected point of 137Cs
<0.01 0.01-0.02 0.02-0.05 0.05-0.1 0.1-0.19 0.19-0.38 0.38-0.95 0.95-1.9 1.9-3.8 3.8-9.5 μSv/h
<0.05 0.05-0.1 0.1-0.25 0.25-0.5 0.5-1 1-2 2-5 5-10 10-20 20-50 mSv/y

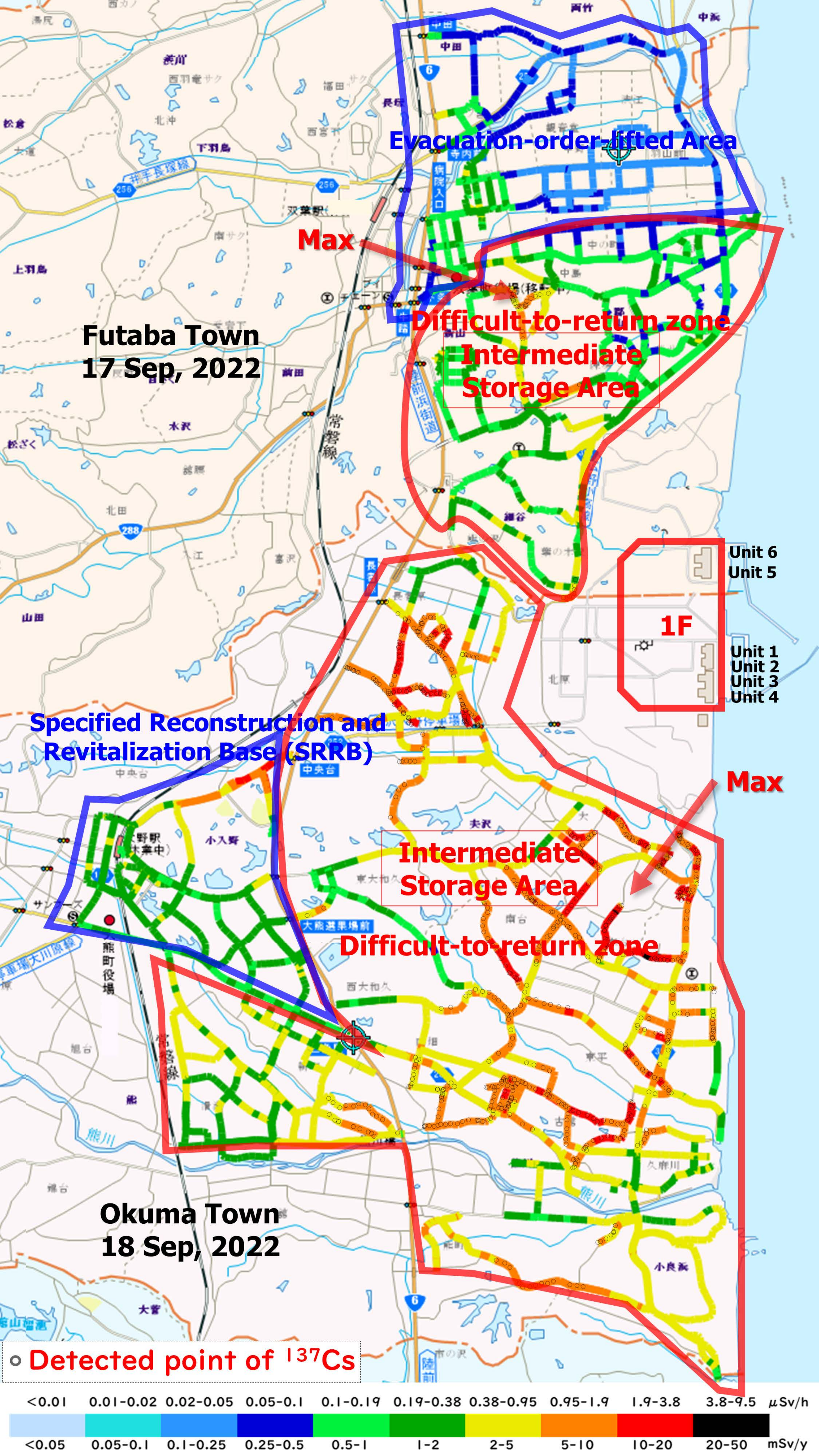

Evacuation-order-lifted Area
Max
Difficult-to-return zone
Intermediate Storage Area
Futaba Town
17 Sep, 2022
Unit 6
Unit 5
1F
Unit 1
Unit 2
Unit 3
Unit 4
Specified Reconstruction and Revitalization Base (SRRB)
Max
Intermediate Storage Area
Difficult-to-return zone
Okuma Town
18 Sep, 2022
Detected point of 137Cs
<0.01 0.01-0.02 0.02-0.05 0.05-0.1 0.1-0.19 0.19-0.38 0.38-0.95 0.95-1.9 1.9-3.8 3.8-9.5 μSv/h
<0.05 0.05-0.1 0.1-0.25 0.25-0.5 0.5-1 1-2 2-5 5-10 10-20 20-50 mSv/y

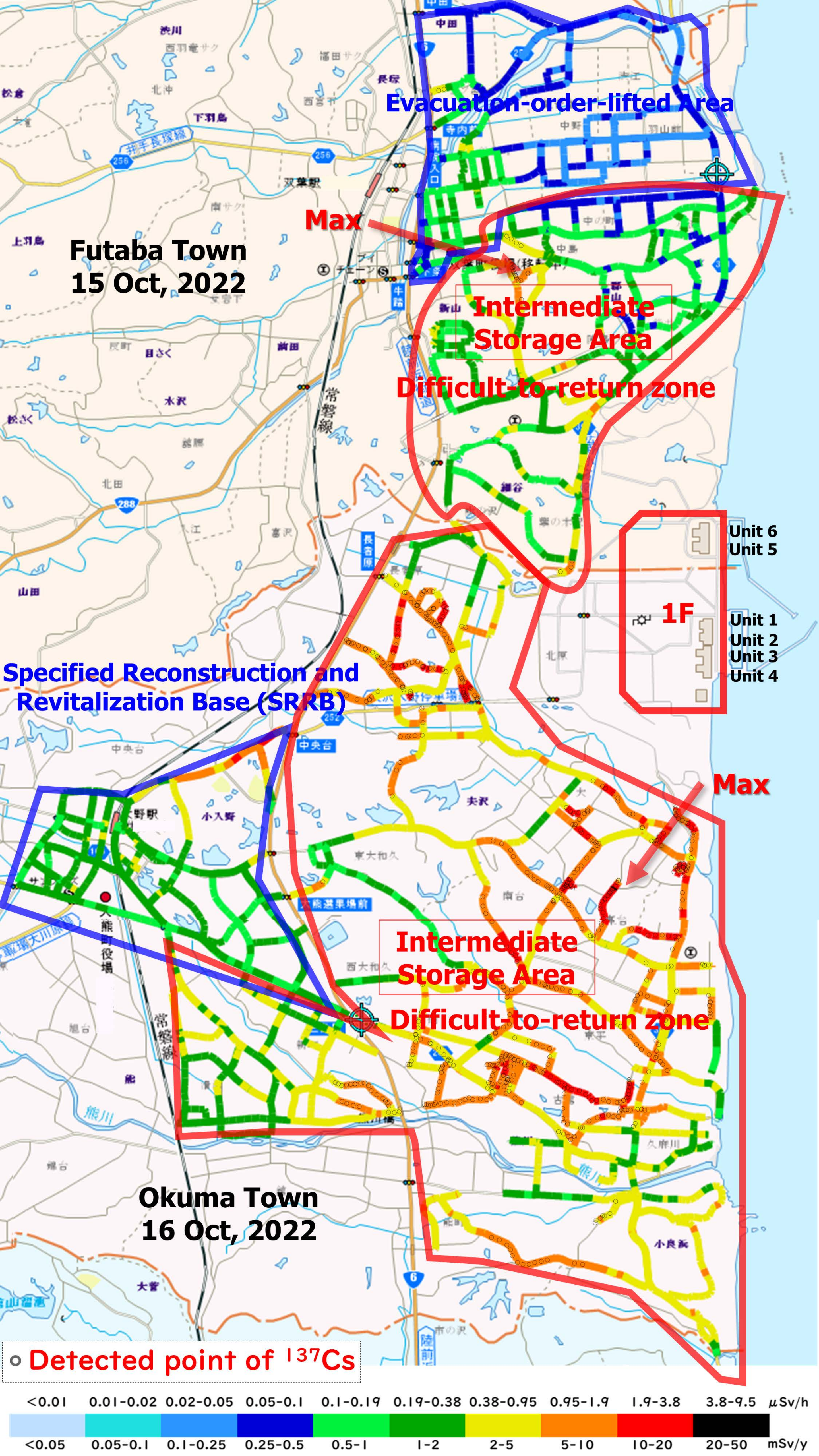

Evacuation-order-lifted Area
Max
Futaba Town
15 Oct, 2022
Intermediate Storage Area
Difficult-to-return zone
Unit 6
Unit 5
1F
Unit 1
Unit 2
Unit 3
Unit 4
Specified Reconstruction and Revitalization Base (SRRB)
Max
Intermediate Storage Area
Difficult-to-return zone
Okuma Town
16 Oct, 2022
Detected point of ^{137}Cs
<0.01 0.01-0.02 0.02-0.05 0.05-0.1 0.1-0.19 0.19-0.38 0.38-0.95 0.95-1.9 1.9-3.8 3.8-9.5 μSv/h
<0.05 0.05-0.1 0.1-0.25 0.25-0.5 0.5-1 1-2 2-5 5-10 10-20 20-50 mSv/y

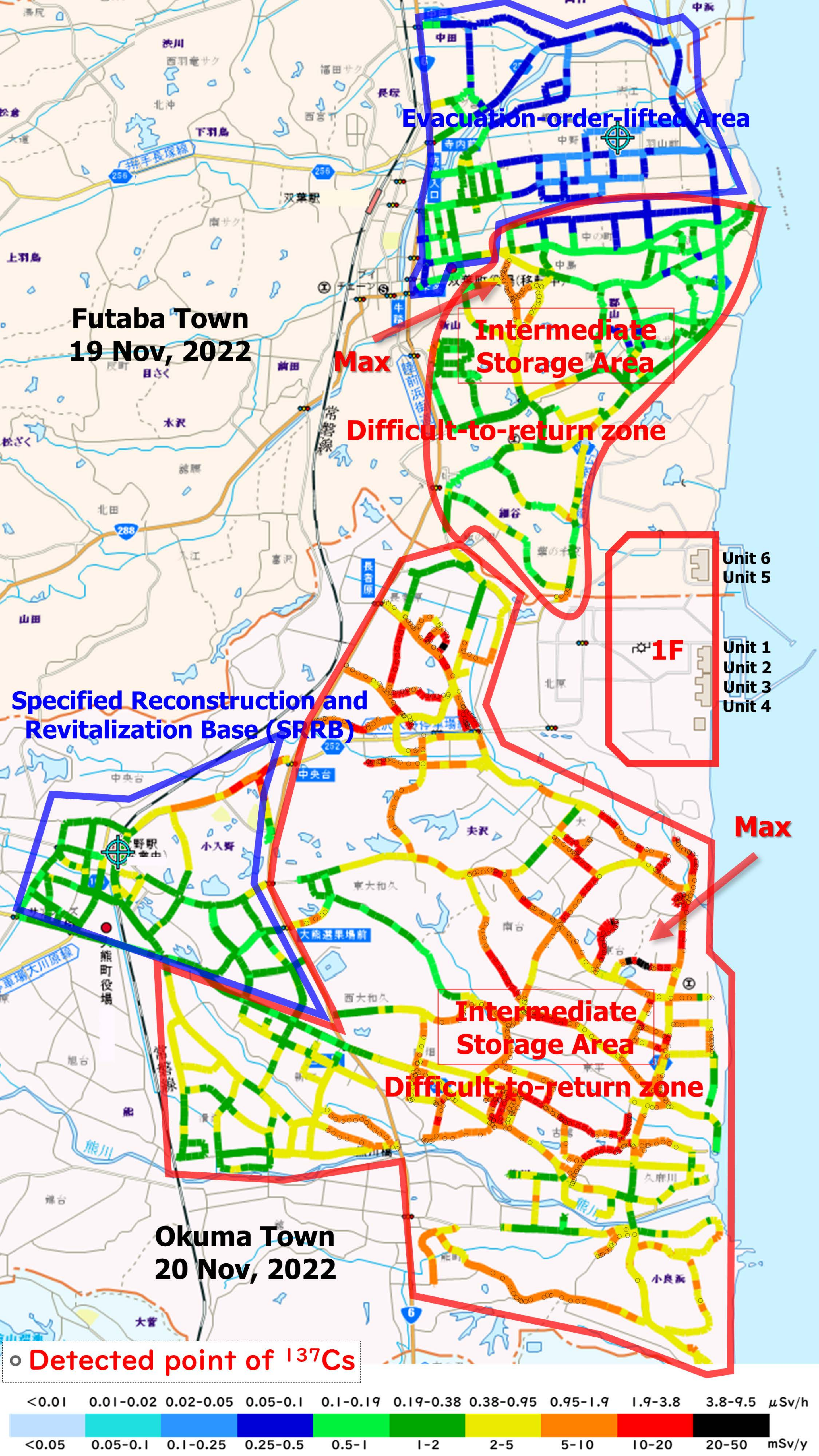
Evacuation-order-lifted Area
Futaba Town
19 Nov, 2022
Max
Intermediate
Storage Area
Difficult-to-return zone
Unit 6
Unit 5
1F
Unit 1
Unit 2
Unit 3
Unit 4
Specified Reconstruction and
Revitalization Base (SRRB)
Max
Intermediate
Storage Area
Difficult-to-return zone
Okuma Town
20 Nov, 2022
Detected point of 137Cs
<0.01 0.01-0.02 0.02-0.05 0.05-0.1 0.1-0.19 0.19-0.38 0.38-0.95 0.95-1.9 1.9-3.8 3.8-9.5 μSv/h
<0.05 0.05-0.1 0.1-0.25 0.25-0.5 0.5-1 1-2 2-5 5-10 10-20 20-50 mSv/y

Maximum points of ambient dose rate

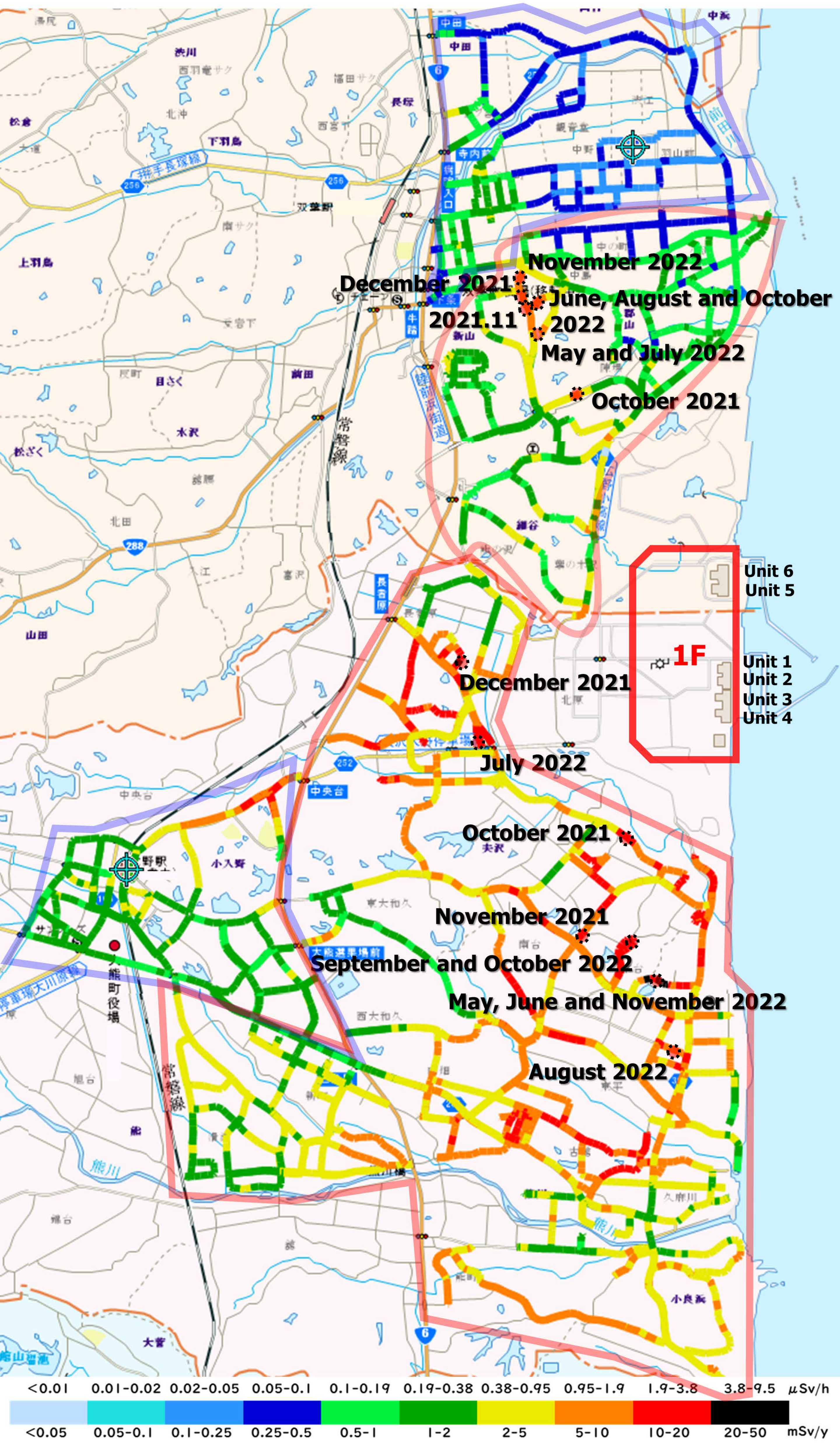

Figure S1. Real-time map of color-scaled ambient dose rates and detected points of 137Cs among sampling locations in the interim storage facility sites in the difficult-to-return zone in Futaba town and Okuma Town. The data were collected using a Radi-probe car-borne survey system during October 2021 to November 2022. The radiation map was modified by the second author (YT) using GIS and PowerPoint software, based on the Radi-probe mapping data (GIS software: Shobunsha Publications, Inc., Tokyo, Japan. https://www.mapple.co.jp/en/; the Radi-probe system: Chiyoda Technology Corp., Tokyo, Japan. http://www.c-technol.co.jp/eng). Map reprinted from the map software (Mapple, ver. 20) for the Radi-probe system under a CC BY license, with permission (No. 4-063) from Shobunsha Publications, Inc., Tokyo, Japan; original copyright 2019 and Chiyoda Technology Corp., Tokyo, Japan.
