# Supplementary material for: Temporal variation in environmental radioactivity and radiation exposure doses in the restricted areas around the Fukushima Daiichi Nuclear Power Plant
Source: Sci Rep. 2023 Dec 18;13:22459. doi: 10.1038/s41598-023-49821-8 (PMC10725873; doi:10.1038/s41598-023-49821-8)
Supplement: Supplementary file 3 — Supplementary Information 3. [file 41598_2023_49821_MOESM3_ESM.docx]

**Supplementary information**

Physical decay of radiocesium from October 2021 to November 2022. Effective doses for workers and residents in Futaba town, Okuma town, and Tomioka town. Real-time map of color-scaled ambient dose rate and ^137^Cs detection points among the sampling locations in the ISF sites in the DRZs in Futaba town and Okuma town from October 2021 to November 2022.
